# The Relationship between Alexithymia and General Symptoms of Patients with Depressive Disorders

**DOI:** 10.4306/pi.2008.5.3.179

**Published:** 2008-09-30

**Authors:** Ju Hee Kim, Seung Jae Lee, Hyo Deog Rim, Hea Won Kim, Geum Ye Bae, Sung Man Chang

**Affiliations:** Department of Psychiatry, School of Medicine, Kyungpook National University, Daegu, Korea.

**Keywords:** Alexithymia, Depression, Symptomatology, Toronto Alexithymia Scale-20, Symptom Checklist-90-Revised

## Abstract

**Objective:**

Depression has been associated with alexithymic features. However, few studies have investigated the differences in the general symptoms of patients with depressive disorders according to the presence of alexithymia. Thus, the aim of this study was to evaluate the relationship between alexithymia and symptoms experienced by patients with clinically diagnosed depressive disorders.

**Methods:**

A chart review of patients who were evaluated using the Korean version of the 20-item Toronto Alexithymia Scale (TAS-20) and Symptom Checklist 90-Revised (SCL-90-R) at the same time between the years 2003 and 2007 was conducted. A total of 104 patients with depressive disorders were included and divided into two groups: alexithymia (n=52) and non-alexithymia (n=52). A direct comparison between the two groups was carried out. Regression analysis was also carried out for the TAS-20 total and subset scores in order to model the relationship between alexithymia and symptoms.

**Results:**

The presence of alexithymia was confirmed in 50% of the patients with depressive disorders, and the symptoms of depressive patients with alexithymia were more severe than those of their non-alexithymic counterparts on all 9 symptom domains of the SCL-90-R. Furthermore, regression analysis revealed that the presence of alexithymia was positively associated with depression, phobic anxiety, and psychoticism but inversely associated with anxiety.

**Conclusion:**

These results suggest that the clinical features of depression are partially dependent on the presence of alexithymia. Alexithymic patients with depressive disorders are likely to show more severe depressive, psychotic, and phobic symptoms. In other words, clinicians should suspect the presence of alexithymic tendencies if these symptoms coexist in patients with depressive disorders and address their difficulties in effective communication.

## Introduction

Alexithymia, a term originally derived from Latin, is the inability to describe emotions with words. Sifneos, who formerly defined the clinical features of alexithymia, positioned this concept in a theoretical framework with Nemiah in 1970.^1^,^2^ Alexithymia is a multidimensional personality construct characterized by three core features: a) difficulty identifying emotions and distinguishing between emotions and bodily sensations, b) difficulty describing or communicating emotions to others, and c) an externally oriented style of thinking.^3^ Alexithymia has important clinical implications, as it not only creates interpersonal problems but also limits the responsiveness of those having alexithymic tendencies to psychotherapy. Therefore, early identification and modification of alexithymic tendencies are necessary to improve the therapeutic relationship and, consequently, treatment outcome.

To date, depression has been identified as the psychiatric feature most consistently associated with alexithymia. Previous literature has indicated that there is a strong association between depression and alexithymia in both depressive patients^4^-^6^ and general populations.^7^-^9^ Longitudinal studies in patients with depressive disorder also showed that recovery from depression was associated with a decrease in alexithymic features.^4^,^6^ Several studies have revealed a positive relationship between depression and the level of difficulty identifying and describing feelings. However, in general, the lack of an imaginative capacity and externally oriented thinking style are not related to depression.^10^-^12^ One prospective study showed that difficulties in identifying and describing feelings are associated with changes in mood, while externally oriented thinking is not.^6^

In the clinical world, patients with depressive disorders do not solely suffer from psychiatric symptoms measured by the Beck Depression Inventory (BDI), which was used in most studies demonstrating the relationship between depression and alexithymia. For instance, psychotic features are estimated to be present in approximately 12% to 25% of patients with depressive disorders.^13^,^14^ Anxiety symptoms are also common in patients with major depression: 62% present with psychic anxiety, 42% have somatic anxiety, and 29% have panic attacks.^15^ However, since most of the previous studies regarding the relationship between depressive disorder and alexithymia only used the BDI to evaluate depressive disorder, the relationship between general symptoms and alexithymia in patients with depressive disorder has scarcely been studied. Honkalampi et al. reported that alexithymic patients with depressive disorders showed more overall psychopathology (anxiety, hostility, psychotic symptoms, and somatization) and severe depression than their nonalexithymic counterparts.^5^ Grabe et al. revealed that a broad range of current psychopathology is associated with difficulties identifying feelings in subjects with 9 different psychiatric disorders.^16^ However, the meanings of these two studies were limited in that the former was conducted in inhomogeneous psychiatric patients, and the latter did not consider the dimensions of alexithymia.

Therefore, the aim of this study was to evaluate the relationship between alexithymia and the general symptoms experienced by patients with depressive disorders. We hypothesized that patients with depressive disorders and alexithymia would have different symptoms and manifestations than patients with depression but no alexithymia and that a certain dimension of alexithymia would be related to a specific symptom.

## Methods

### Subjects

This study included 104 patients diagnosed with depressive disorders. Sixty-two of the patients were male and 42 were female, and their mean age was 39.9±16.1 years (range, 18-76 years). The primary diagnosis was major depressive disorder in 40 patients, dysthymic disorder in 35 patients, and depressive disorder not otherwise specified (NOS) in 29 patients. The mean Clinical Global Impression (CGI) score was 3.5±1.0, which was indicative of mild to moderate illness. Only 12 patients were not being treated with psychiatric medication; 86 patients were taking antidepressants at therapeutic dosages, most of whom (n=71) were also taking anxiolytic medications; two patients were only taking antipsychotic medications, and two patients were taking only anxiolytic medications. Table 1 shows the demographic and clinical characteristics of the sample.

### Procedures

All patients met the Diagnostic and Statistical Manual of Mental Disorders, fourth edition (DSM-IV) criteria for a primary diagnosis of a depressive disorder and were evaluated in a single session using the Korean version of the 20-item Toronto Alexithymia Scale (TAS-20K)^17^ and the Symptom Checklist 90-Revised (SCL-90-R)^18^ at Kyungpook National University Hospital, Daegu, Korea, between January 2003 and March 2007. Detailed chart reviews were also conducted for all participants. The exclusion criteria included age under 18 years, mental retardation, and any neurological condition that might affect self-reporting.

Data on demographic and psychosocial factors (i.e., sex, age, and level of education), clinical profiles (i.e., primary and comorbid psychiatric diagnoses present during the evaluation according to DSM-IV criteria, duration of illness, medications and CGI scores), and results of psychological assessments (TAS-20K, SCL-90-R) were analyzed.

Two experienced psychiatrists (K.J.H., K.H.W.) reviewed the patients' charts, newly confirmed the previous diagnosis of depressive disorders, and rated their CGI scores at the time the TAS-20 was conducted based on chart review. Ninety-nine patients (95%) completed these assessments at the time of their first psychiatric visit. This study was approved by the institutional review board of Kyungpook National University Hospital.

### Psychological scales

#### The 20-item Toronto Alexithymia Scale

The TAS-20 has become the most widely used measure of alexithymia.^3^ This self-report questionnaire measures three intercorrelated dimensions of the alexithymia construct: a) difficulties identifying feelings, b) difficulties describing feelings, and c) externally oriented thinking. Each TAS-20 item was rated on a 5-point Likert scale, with total scores ranging from 20 to 100. The cut-off point for alexithymia was ≥61.^19^ The study by Lee and colleagues^17^ provided a detailed report concerning the translation of the TAS-20 into Korean. Using confirmatory factor analysis, Lee et al. showed that the three-factor structure of the original scale is consistent with the Korean version of the scale (Cronbach's alpha=0.76).

#### Symptom Checklist-90-Revised

The SCL-90-R is one of the most widely used and well-validated self-report symptom inventories designed to evaluate a broad range of psychological problems and symptoms of psychopathology.^20^ Respondents rate 90 items using a 5-point scale (1="no problem" to 5="very serious") to measure the extent to which they have experienced the listed symptoms in the past 7 days. It consists of 9 symptom scales (Somatization, Obsessive-Compulsive, Interpersonal Sensitivity, Depression, Anxiety, Hostility, Phobic Anxiety, Paranoid Ideation, and Psychoticism) and 3 global indices [Global Severity Index (GSI), Positive Symptom Distress Index (PSDI), and Positive Symptom Total (PST)]. Higher scores on the SCL-90-R are indicative of greater psychological distress. The internal consistency (coefficient alphas) for the nine symptom dimensions ranged from 0.77 for psychoticism, to a high of 0.90 for depression. The Korean version of the SCL-90-R was used in this study.^18^

#### Clinical Global Impression-Severity Scale

The CGI-Severity (CGI-S)^21^ was used to assess the clinical impression of the current state of the patient's illness. The rater was asked to "consider his total clinical experience with the given population." The time span considered is the week prior to the rating; and the following scores can be given: 1=normal, not at all ill; 2=borderline mentally ill; 3=mildly ill; 4=moderately ill; 5=markedly ill; 6=severely ill; and 7=among the most extremely ill patients.

### Statistical analysis

Based on their TAS-20 scores, the subjects were divided into two groups, alexithymia (TAS-20K total score ≥61) and non-alexithymia (TAS-20K total score <61). Between-group comparisons were performed using the chi-square test for categorical variables and independent t-test for continuous variables. To identify certain symptoms associated with alexithymia, multiple linear regression analysis was used to model the relationship between alexithymia and symptoms. Age, education level, duration of illness, and the presence of comorbid psychiatric disorder were also included to adjust for confounding effects. Regression analyses were carried out for total score and score on each of the three subfactors in the TAS-20.

Statistic analyses were performed using SPSS software (version 12.0; SPSS Inc., Chicago, IL) All significant levels were two-tailed and set at a 0.05 significance level.

## Results

### Between-group comparisons

The only significant difference between the alexithymia and non-alexithymia groups was the ratio of comorbid psychiatric disorder (t=5.0, p=0.026) to duration of illness (t=0.2, p=0.040). There were no significant group differences in the demographic and clinical characteristics of the patients. The patients with depressive disorders and alexithymia showed a significant increase on all 9 of the items on the SCL-90-R compared to those without alexithymia (all ps<0.001)(Table 2).

### Multiple linear regression analysis

Multiple linear regression analysis was carried out using the simultaneous entry method. In terms of total score on the TAS-20, this analysis produced a value of 0.695 for R^2^, indicating that approximately 70% of the variance was accounted for by the independent variables (F_13,90_=15.77, p<0.001; adjusted R^2^=0.651). After adjusting for possible confounders, total TAS-20 score was positively associated with depression, phobic anxiety, and psychoticism and inversely associated with anxiety, among the 9 dimensions of the SCL-90-R. Factor 1 was associated with somatization and showed a trend towards association with psychoticism. Factor 2 was positively associated with depression and negatively associated with anxiety. Factor 3 was positively associated with psychoticism and negatively associated with anxiety (Table 3).

## Discussion

In this study, we explored the relationship between alexithymia and the general symptoms of patients with depressive disorders. The presence of alexithymia was confirmed in 50% of patients, and the alexithymic patients with depressive disorders showed more severe symptoms in all 9 symptom domains of the SCL-90-R than their nonalexithymic counterparts. Furthermore, regression analysis revealed that the presence of alexithymia was positively associated with depression, phobic anxiety, and psychoticism, but inversely associated with anxiety.

Fifty-two (50%) of the 104 patients with depressive disorders were considered alexithymic, and the patients' scores on the SCL-90-R revealed a greater incidence of psychopathology on all 9 SCL-90-R subscales in alexithymic patients than in nonalexithymic patients. These findings are generally consistent with those of a previous study showing that alexithymic patients with depressive disorders had a greater incidence of comorbid psychiatric disorder than their nonalexithymic counterparts.^5^ Thus, these findings suggest that, even among depressive patients, the presence of alexithymic tendencies is related to more severe psychopathologies. It is important to note that there were no between-group differences in CGI score and the proportion of depressive diagnoses.

The symptom of depression, measured by the SCL-90-R, was positively associated with TAS-20 total score and factor 2 (difficulties describing feelings) in this study. By definition, the depression item includes loss of energy, diminished interest and suicidal ideation, and these are consistent with the manifestations of depressive disorders. These findings mean that, even in patients with depressive disorders, the more severe the alexithymia, the more severe the depression. Furthermore, these findings corroborate the observation of Honkalampi et al.^5^ More precisely, Sayar et al.^22^ showed that individuals with alexithymic characteristics who had difficulty distinguishing feelings from bodily sensations displayed more suicidal ideation related to hopelessness.

In particular, subjects having difficulties describing their feelings were found to suffer from more depressive symptoms in this study. Duddu et al.^11^ reported that patients with depressive disorder had greater difficulty describing their feelings when compared to subjects with somatoform disorder. Bankier et al.^10^ also showed that depression was significantly associated with factor 2. The results of previous studies and ours indicated that factor 2 in the alexithymic construct may be more specifically related to depressive symptoms.

Another item that was associated with alexithymia in this study was the psychoticism domain, which showed a positive relationship with total TAS-20 score and factor 3 (externally oriented thinking). This dimension includes isolation, withdrawal, schizoid lifestyle, hallucination and thought broadcasting. When compared with the paranoid ideation domain, it seems that psychoticism reflects behavioral (i.e., negative psychotic features) or perceptual symptoms rather than the content of thought shown in paranoid ideation. Nkam et al. observed that negative schizophrenic patients had significantly higher total scores in alexithymia than those with positive symptoms,^23^ and these results seem to agree with our research findings. More importantly, factor 3 was significantly associated with psychoticism. Factor 3, which is decomposed into two factors, pragmatic thinking and lack of subjective significance or importance of emotions, is related to constricted imaginal processes, as evidenced by a paucity of fantasies.

Thus, factor 3 may be closely related to anhedonia, poverty of thought, and alogia, which are also characteristics of the negative symptoms of schizophrenia. We assumed that this is the reason why factor 3 was specifically associated with psychoticism. In this context, some studies concluded that a deficit in the ability to experience emotions may contribute to interpersonal or social isolation, limited social engagement, and other impoverished environments.^24^,^25^ However, there were also several studies that failed to show a relationship between alexithymia and negative symptoms.^26^,^27^

Interestingly, the phobic anxiety dimension of the SCL-90-R was positively associated with TAS-20 total score while the anxiety dimension was inversely correlated with TAS-20 total score, factor 2 (difficulties describing feelings) and factor 3 (externally oriented thinking). Anxiety includes general signs of anxiety such as nervousness, tension and trembling, while phobic anxiety is defined as a persistent fear response that is irrational and disproportionate to the stimulus and is very similar in definition to agoraphobia. Although Devine et al.^28^ observed that the degree of difficulty in recognizing and identifying feelings corresponded to the anxiety level of individuals, there was a negative relationship between the difficulty in communicating feelings and the intensity of anxiety symptoms in other studies.^22^,^29^ Based on the findings of previous studies and ours, we speculate that, as anxiety increases to a certain extent, it makes individuals feel dystonic to their symptoms (internally oriented thinking), and they therefore overly recognize and describe their feelings. In effect, individuals who experience difficulty in communicating their feelings avoid relationships using various avoidance/escape methods, and thereby decrease their anxiety level.^22^,^29^ Once anxiety surges to a degree of phobic state, people would be confused about their emotional state and make frantic efforts to avoid a given situation.

The 'Somatization' category of the SCL-90-R was only associated with factor 1 (difficulties identifying feelings). Emotional deficits of alexithymic depressive patients underlie failures in the capacity to recognize physical sensations as the somatic manifestations of emotions. Consequently, choosing not to deal with the emotions that underlie these somatic sensations results in somatosensory amplification, which may then be misinterpreted as physical illness.^19^,^30^ Our study findings also supported these interpretations. Due to their difficulty experiencing emotions, alexithymic patients with depressive disorders are thought to focus on somatic manifestations of emotional arousal, resulting in amplification of bodily sensations and misinterpretation of somatic sensations as signs of physical illness.^30^

The limitations of the present study are as follows. The major limitation of this study is its cross-sectional nature, which makes a causal inference impossible. Second, although we thoroughly reviewed data and discarded improper data, some data such as diagnosis and disease severity could be biased due to the retrospective study design. Finally, the sample population was comprised of patients from Kyungpook National University Hospital, which makes it difficult to generalize this study to the general psychiatric population, and a selection bias may have occurred because more severe patients tend to be referred to university hospitals in Korea.

In conclusion, the results of the present study indicate that the clinical features of depression partially depend on the presence of alexithymia. Alexithymic patients with depressive disorders are likely to show more severe depressive, psychotic, and phobic symptoms. In other words, clinicians should suspect the presence of alexithymic tendencies if these symptoms coexist in patients with depressive disorders, and consider specific psychotherapeutic techniques for improving affect differentiation in order to enhance effective communication.

## Figures and Tables

**TABLE 1:**
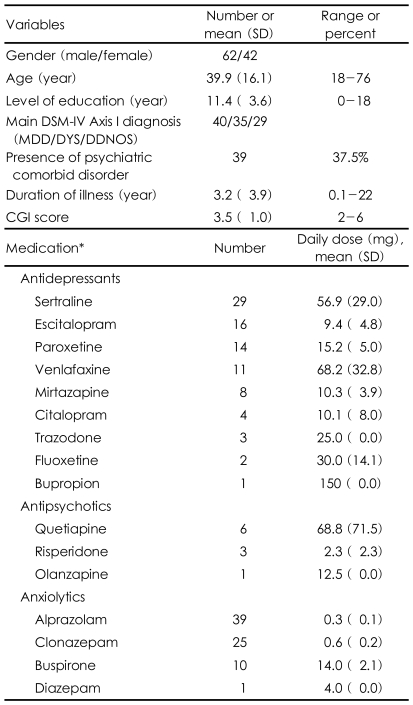
Demographic and clinical characteristics of patients with depressive disorders (N=104)

^*^12 patients were not taking any psychiatric medications at the time of their psychological evaluation. SD: standard deviation, DSM-IV: Diagnostic and Statistical Manual of Mental Disorders, fourth edition, MDD: major depressive disorder, DYS: dysthymic disorder, DDNOS: depressive disorder not otherwise specified, CGI: Clinical Global Impression

**TABLE 2:**
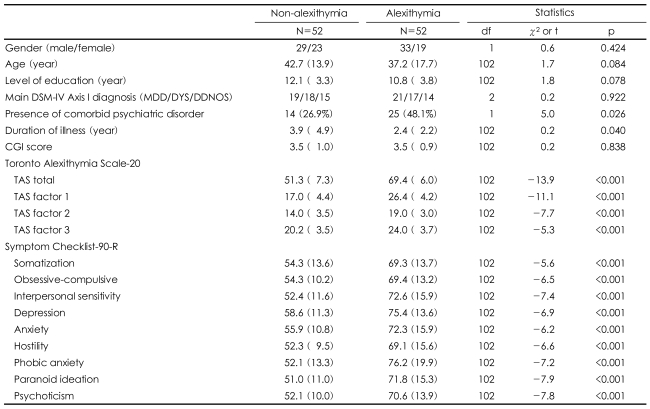
Comparison between non-alexithymia and alexithymia group among patients with depressive disorder

Figures in parenthesis represent standard deviations except one variable of the presence of comorbid psychiatric disorder. DSM-IV: Diagnostic and Statistical Manual of Mental Disorders, fourth edition, MDD: major depressive disorder, DYS: dysthymic disorder, DDNOS: depressive disorder not otherwise specified, CGI: Clinical Global Impression, TAS: Toronto Alexithymia Scale

**TABLE 3:**
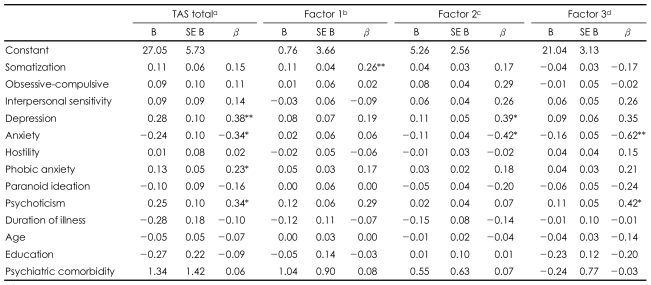
Relationships of TAS-20 total scores and subfactors with 9 symptom dimensions (simple regression analyses)

a: R^2^=0.695 (p<0.01), b: R^2^=0.613 (p<0.001), c: R^2^=0.530 (p<0.001), d: R^2^=0.313 (p=0.001). ^*^p<0.05, ^**^p<0.01. TAS: Toronto Alexithymia Scale

## References

[1] Nemiah JC, Sifneos PE (1970). Psychosomatic illness: a problem in communication. Psychother Psychosom.

[2] Sifneos PE (1996). Alexithymia: past and present. Am J Psychiatry.

[3] Bagby RM, Parker JD, Taylor GJ (1994). The twenty-item Toronto Alexithymia Scale--I. Item selection and cross-validation of the factor structure. J Psychosom Res.

[4] Honkalampi K, Hintikka J, Laukkanen E, Lehtonen J, Viinamäki H (2001). Alexithymia and depression: a prospective study of patients with major depressive disorder. Psychosomatics.

[5] Honkalampi K, Saarinen P, Hintikka J, Virtanen V, Viinamäki H (1999). Factors associated with alexithymia in patients suffering from depression. Psychother Psychosom.

[6] Saarijärvi S, Salminen JK, Toikka TB (2001). Alexithymia and depression: a 1-year follow-up study in outpatients with major depression. J Psychosom Res.

[7] Hintikka J, Honkalampi K, Lehtonen J, Viinamäki H (2001). Are alexithymia and depression distinct or overlapping constructs?: a study in a general population. Compr Psychiatry.

[8] Honkalampi K, Hintikka J, Tanskanen A, Lehtonen J, Viinamäki H (2000). Depression is strongly associated with alexithymia in the general population. J Psychosom Res.

[9] Honkalampi K, Koivumaa-Honkanen H, Tanskanen A, Hintikka J, Lehtonen J, Viinamäki H (2001). Why do alexithymic features appear to be stable? A 12-month follow-up study of a general population. Psychother Psychosom.

[10] Bankier B, Aigner M, Bach M (2001). Alexithymia in DSM-IV disorder: comparative evaluation of somatoform disorder, panic disorder, obsessivecompulsive disorder, and depression. Psychosomatics.

[11] Duddu V, Isaac MK, Chaturvedi SK (2003). Alexithymia in somatoform and depressive disorders. J Psychosom Res.

[12] Haviland MG, Shaw DG, Cummings MA, MacMurray JP (1988). Alexithymia: subscales and relationship to depression. Psychother Psychosom.

[13] Ohayon MM, Schatzberg AF (2002). Prevalence of depressive episodes with psychotic features in the general population. Am J Psychiatry.

[14] Coryell W, Pfohl B, Zimmerman M (1984). The clinical and neuroendocrine features of psychotic depression. J Nerv Ment Dis.

[15] Fawcett J, Kravitz HM (1983). Anxiety syndromes and their relationship to depressive illness. J Clin Psychiatry.

[16] Grabe HJ, Spitzer C, Freyberger HJ (2004). Alexithymia and personality in relation to dimensions of psychopathology. Am J Psychiatry.

[17] Lee YH, Rim HD, Lee JY (1996). Development and validation of a Korean version of the 20-item Toronto Alexithymia Scale (TAS-20K). J Korean Neuropsychiatr Assoc.

[18] Kim KI, Kim JH, Won HT (1984). Korean manual of Symptom Checklist-90-Revision.

[19] Taylor GJ, Bagby RM, Parker JDA (1997). Disorders of affect regulation: Alexithymia in Medical and Psychiatric Illness.

[20] Derogatis LR, Rickels K, Rock AF (1976). The SCL-90 and the MMPI: a step in the validation of a new self-report scale. Br J Psychiatry.

[21] Guy W (1976). ECDEU assessment manual for psychopharmacology- revised: NIMH publ.

[22] Sayar K, Ak I (2001). The predictors of somatization: a review. Bull Clin Psychophar.

[23] Nkam I, Langlois-Thery S, Dollfus S, Petit M (1997). Negative symptoms, depression, anxiety and alexithymia in DSM III-R schizophrenic patients. Encephale.

[24] Stanghellini G (1995). Language capacity in paranoid and non-paranoid schizophrenics: a revision of previous findings through the analysis of a larger sample. Neurol Psychiatr Brain Res.

[25] Stanghellini G, Ricca V (1995). Alexithymia and schizophrenias. Psychopathology.

[26] Maggini C, Raballo A, Pelizza L, Paini M, Croci R (2003). Subjective experience of language impairment and psychopathology in schizophrenia. Psychopathology.

[27] Todarello O, Porcelli P, Grilletti F, Bellomo A (2005). Is alexithymia related to negative symptoms of schizophrenia? A preliminary longitudinal study. Psychopathology.

[28] Devine H, Stewart SH, Watt MC (1999). Relations between anxiety sensitivity and dimensions of alexithymia in a young adult sample. J Psychosom Res.

[29] Berthoz S, Consoli S, Perez-Diaz F, Jouvent R (1999). Alexithymia and anxiety: compounded relationships? A psychometric study. Eur Psychiatry.

[30] Taylor GJ, Parker JD, Bagby RM, Acklin MW (1992). Alexithymia and somatic complaints in psychiatric out-patients. J Psychosom Res.

